# Highly Ordered TiO_2_ Nanotube Arrays with Engineered Electrochemical Energy Storage Performances

**DOI:** 10.3390/ma14030510

**Published:** 2021-01-21

**Authors:** Wangzhu Cao, Kunfeng Chen, Dongfeng Xue

**Affiliations:** 1 State Key Laboratory of Crystal Materials, Institute of Crystal Materials, Shandong University, Jinan 250100, China; caowangzhu@sdu.edu.cn; 2 Shenzhen Institute of Advanced Electronic Materials, Shenzhen Institutes of Advanced Technology, Chinese Academy of Sciences, Shenzhen 518055, China

**Keywords:** TiO_2_, crystal growth, Li-ion batteries, nanotube array, anodization

## Abstract

Nanoscale engineering of regular structured materials is immensely demanded in various scientific areas. In this work, vertically oriented TiO_2_ nanotube arrays were grown by self-organizing electrochemical anodization. The effects of different fluoride ion concentrations (0.2 and 0.5 wt% NH_4_F) and different anodization times (2, 5, 10 and 20 h) on the morphology of nanotubes were systematically studied in an organic electrolyte (glycol). The growth mechanisms of amorphous and anatase TiO_2_ nanotubes were also studied. Under optimized conditions, we obtained TiO_2_ nanotubes with tube diameters of 70–160 nm and tube lengths of 6.5–45 μm. Serving as free-standing and binder-free electrodes, the kinetic, capacity, and stability performances of TiO_2_ nanotubes were tested as lithium-ion battery anodes. This work provides a facile strategy for constructing self-organized materials with optimized functionalities for applications.

## 1. Introduction

Since titanium dioxide (TiO_2_) was first used in the electrochemical photolysis of water in 1972, researchers have developed a strong interest in TiO_2_ [1,2,3,4]. TiO_2_ exhibits rich physical and chemical properties such as non-toxicity, high corrosion resistance, biocompatibility, and unique optoelectronic properties, allowing it to maintain good competitiveness in photocatalysis, sensors, dye-sensitized solar cells, and electrochemical energy storage, etc. [5,6,7,8,9,10,11]. After decades of development, nanoscale materials (0D, 1D, 2D, and 3D) are becoming key in controlling various performances [12]. Among these nanomaterials, 1D nanotubes have received more and more attention in the materials field. After the synthesis of carbon nanotubes, the research on highly ordered nanotube structures aroused a great upsurge of interest [13,14,15]. Research on TiO_2_ nanotubes has also became a hot spot.

Three methods can be used for preparing TiO_2_ nanotubes: the template method [16], the hydrothermal (solvothermal) method [17], and the electrochemical anodization method [18]. For the template method, TiO_2_ nanotubes have a larger inner diameter and thicker tube wall, and their morphology is restricted by the template. With the hydrothermal (solvothermal) way, TiO_2_ nanotubes have small tube diameters, thin tube walls, and their morphology is also difficult to control. Of these methods, the anodic oxidation method displays the simplest operation process and has the advantages of vertical arrangement and highly ordered nanotube arrays [19,20]. The preparation technology of anodized TiO_2_ nanotube arrays can be roughly divided into three generations: (i) hydrofluoric acid aqueous electrolyte was used in the first generation, and the length of obtained nanotubes was only 500 nm [21]; (ii) F^−^-containing water-based electrolyte was applied, and the length of the nanotubes was 5 μm [22]; (iii) F^−^-containing organic electrolytes were used, and the length of the nanotubes reached 100–1000 μm, while the nanotubes were smooth and better than those of the previous two generations [23].

Numerous research efforts have been made to study the influencing factors of self-ordered TiO_2_ nanotube structures; i.e., F^−^ concentration, pH value, water content, type of electrolyte, oxidation voltage, oxidation time, and reaction temperature [24]. The concentration of F^−^ ions must be kept at a proper level to achieve the balance between growth and dissolution of nanotubes [25]. With HF acidic electrolytes, only 5 μm-long nanotubes were produced [19]. In a fluoride salt solution, the chemical dissolution rate of TiO_2_ reduced and 24 μm-long nanotubes can be obtained [23]. F^−^ ions are more aggressive in aqueous solutions than in organic media; typically side walls of the nanotubes appear distorted in water, while they grow more smoothly in organic solutions [26]. The lower water content in organic electrolytes increases the growth rate of the nanotubes [27]. The applied potential affects the migration of ions and the morphology of the nanotubes, which is often 5–30 V in water electrolyte and 10–60 V in organic electrolytes [28]. In organic electrolytes, the optimal temperature range for nanotubes growth was shown to be between 0 and 40 °C [29]. However, there still exist less known but important aspects to be explored, such as the fundamentals of nanotube growth, the effect of defects in the Ti substrate, the effect of heat-treatments, the improvements in the applications of nanotubes, etc.

Vertically oriented self-organized TiO_2_ nanotubes have become an excellent candidate material for lithium-ion battery anodes. The benefits of TiO_2_ nanotube arrays are shown as follows [30,31,32]: (i) Good structural stability, large specific surface area, small volume expansion rate. (ii) With a hollow tubular structure, the gaps between tubes are conducive to the penetration of the electrolyte. The inner and outer walls of the tube increase the contact area between the electrode and the electrolyte, shorten the diffusion path of lithium ions, and facilitate the reversible insertion/extraction of lithium ions. (iii) The active materials are firmly combined with the metal titanium matrix, without the need to add additional binders and conductive agents. (iv) As a negative electrode, the high voltage can avoid the precipitation of metallic lithium, so it is a promising candidate as a safe lithium-ion battery anode material.

In this work, we revisited the so-called “old field” of anodic oxidation and finding an inherent mechanism for engineering regular nanotube structured materials with electrical and chemical fields to improve the areal capacity, rate capability, and cycling stability of lithium-ion battery anodes, and fabrication strategies for TiO_2_ nanotube electrodes were systematically optimized.

## 2. Experimental Section

### 2.1. Reagents and Materials

Titanium foil (99.99%, Qinghe Shenghang Metal Material Co. Ltd., Shanghai, China); platinum electrode (99.99%, Shanghai Yueci Electronic Technology Co. Ltd., Shanghai, China); ethylene glycol (EG, 99.5%, Sinopharm Chemical Reagent Co. Ltd., Shanghai, China); ammonium fluoride (NH_4_F, 96.0%, Tianjin Komiou Chemical Reagent Co. Ltd., Tianjin, China); anhydrous ethanol (99.7%, Tianjin Fuyu Fine Chemical Co. Ltd., Tianjin, China).

### 2.2. Preparation of TiO_2_ Nanotube Arrays

**Figure 1:**
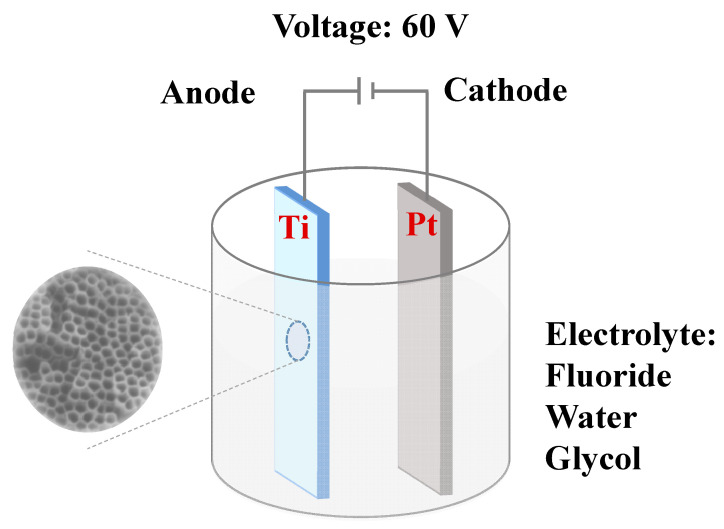
Schematic illustration of an electrochemical anodic oxidation cell.

The high-purity titanium foil (10 mm × 35 mm × 0.1 mm) was ultrasonically cleaned with anhydrous ethanol and deionized water for 10 min, respectively. Then, titanium foil was dried in the air. One side of dried titanium foil was sealed with insulating tape. As shown in Figure 1, a two-electrode system was used, and a high-purity platinum foil (15 mm × 15 mm × 0.1 mm) was used as a counter electrode. The two electrodes had a distance of 2 cm. Electrolytes were prepared with 75 mL EG, 0.5 wt% NH_4_F and 757 μL deionized water. In another set of experiments, 0.2 wt% NH_4_F was used, and other conditions remained unchanged. At 20 °C, a DC power supply (NPS3010W, Wanptek, Shenzhen, China) was used to provide a constant voltage potential of 60 V, and oxidation time was 2, 5, 10, and 20 h, respectively. After reaction, the titanium foil was taken out and cut into 3 sheets of 10 mm × 10 mm. Then, ultrasonic cleaning was conducted with EG and anhydrous ethanol for 10 min, respectively, and we then rinsed the sheets lightly with anhydrous ethanol three times. Finally, we removed the back insulating tape and dried the sheets in the air.

As for the annealing steps, the prepared TiO_2_ nanotube array was first placed in a quartz boat and then transferred to the programmed temperature-controlled muffle furnace. Then, it was held at 450 °C for two hours with a heating rate of 5 °C per minute. After temperature decreased to room temperature, the annealed sample was taken out. The sample name of TiO_2_-5 h-450 °C denoted a sample anodized for 5 h and heated at 450 °C. TiO_2_-5 h denoted a sample anodized for 5 h before annealing.

### 2.3. Electrochemical Analysis of Li/TiO_2_ Cells

In a glove box filled with Ar, the Li/TiO_2_ cells were assembled into 2032 coin batteries. A glass microfiber separator was put between the electrode and the lithium sheet, and the separator was fully wetted with 1 M liquid electrolyte LiPF_6_ dissolved in a 1:1 volume ratio of dimethyl carbonate (DMC) and ethylene carbonate (EC). In this work, the Li/TiO_2_ cells were tested with charge–discharge equipment (Lanhe CT3001A, Wuhan, China) at a series of current densities within the voltage range of 1–3 V (vs. Li/Li^+^). Cyclic voltammograms (CV) and electrochemical impedance spectroscopy were conducted at an electrochemical workstation (CHI 660E, Shanghai, China).

### 2.4. Characterization

The samples were investigated by X-ray diffraction (XRD, Rigaku, SmartLab 9KW, Tokyo, Japan), scanning electron microscopy (SEM, JEOL, JSM-6700F, Tokyo, Japan) and UV laser confocal Raman Spectrometer (Horiba, LabRAM HR Evolution, 532 laser, 100–1000 cm^−1^, Paris, France). The XRD test parameters were: Cu target, Kα λ = 0.154056 nm, the scanning speed was 20° per minute, the scanning step was 0.01°, and the scanning range was 10–90°.

## 3. Results and Discussion

**Figure 2:**
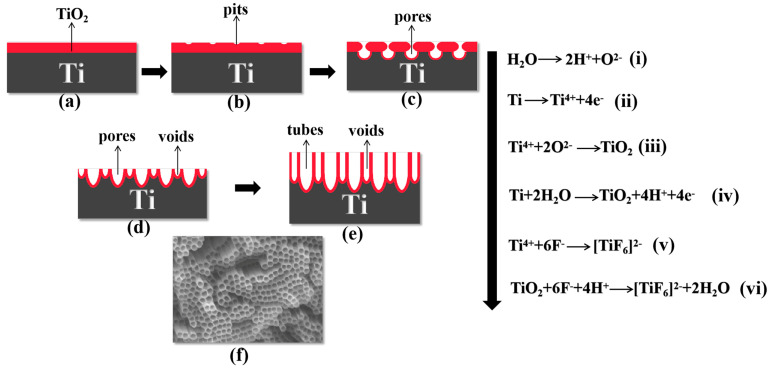
Schematic diagram of nanotube evolution at constant anodization voltage: (**a**) formation of TiO_2_ barrier layer; (**b**) pits formed on the oxide layer; (**c**) pits growing into pores morphology; (**d**) pores’ growth and voids’ formation; (**e**) formation of TiO_2_ nanotubes; (**f**) top view of fully grown TiO_2_ nanotube arrays. Formulas (i)–(vi) are the chemical reactions that occurred during the anodic oxidation process.

**Figure 3:**
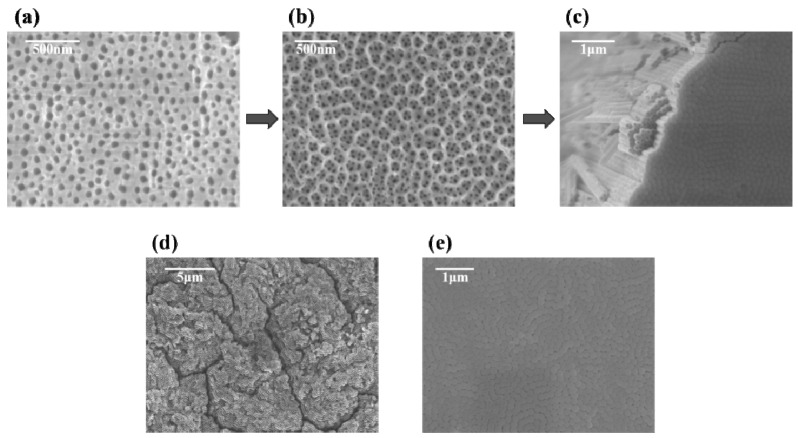
SEM images showing growth process of TiO_2_ nanotube array: (**a**) pits formed on the oxide layer; (**b**) conversion of pits to nanopores; (**c**) conversion of nanopores to nanotubes; (**d**) the top view of the nanotube array; (**e**) the bottom view of the nanotube array.

The process of anodizing and growing TiO_2_ nanotube arrays in F^−^ ions containing organic electrolytes was separated into four stages. **In the first stage**, a layer of dense TiO_2_ barrier layer was rapidly generated at the anode after voltage was applied (Figure 2a). In this stage, H_2_O was ionized into H^+^ cations and O^2−^ anions (Figure 2, Formula (i)). At the same time, metal titanium was dissolved into Ti^4+^ cations (Figure 2, Formula (ii)), and then a large number of Ti^4+^ cations combined with O^2−^ anions to form a dense TiO_2_ film (Figure 2, Formula (iii)). The overall reaction can be summarized as Formula (iv) in Figure 2 [33]. **In the second stage**, F^−^ ions chemically etched the barrier layer to produce a large number of pits (Figure 2b); as the barrier layer continued to penetrate into the titanium substrate, the pits became larger and nanopores appeared (Figure 2c). The formation of a dense oxide layer resulted in volume expansion and generated internal stress. Then, the F^−^ ions reacted with the TiO_2_ barrier layer and chemical etching occurred: the chemical dissolution of oxides (Figure 2, Formula (vi)) or the direct complexation of the free Ti^4+^ cations (Figure 2, Formula (v)). A large number of pits on the oxide layer were found (Figure 3a) and these pits were the precursors of pores (Figure 3b) [34].

**In the third stage**, the porous membrane began to grow steadily and form TiO_2_ nanotubes (Figure 2d,e and Figure 3c). As the pores extended to the titanium substrate, the electric field intensity of the metal region increased, accelerating the growth and dissolution rate of the oxide film. Simultaneously, stress corrosion occurred between adjacent nanopores, which created voids at the interface of the pores. Due to the growth stresses, pores were formed as a consequence of flow of material in the oxide barrier layer under the porous layer toward the wall regions and field-assisted plasticity of the film material [27]. With the nanopores continuing to grow, the joints between pores continued to extend to the titanium substrate, eventually forming the tube wall [35]. The formation of dynamic equilibrium between the oxidation reaction at the oxide/titanium substrate interface (Figure 2, Formula (iv)) and the dissolution reaction at the electrolyte/oxide interface (Figure 2, Formulas (v) and (vi)) led to the development of TiO_2_ nanotubes [36]. The top and bottom of the nanotube array are exhibited in Figure 3d,e. **In the final stage**, when the dissolution rate of TiO_2_ nanotubes on the top was equal to the growth rate of the nanotubes, the reaction entered the equilibrium stage, at which time the length of the nanotubes no longer increased [33].

**Figure 4:**
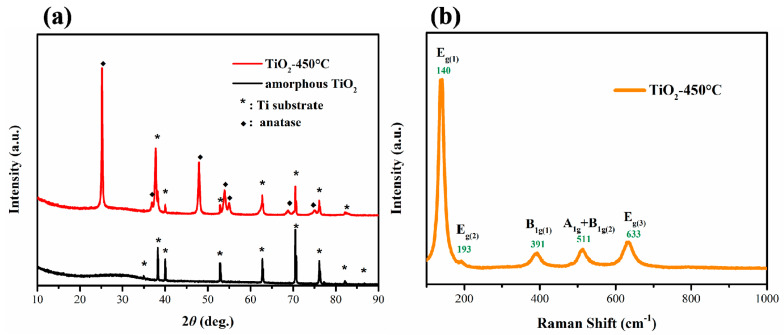
(**a**) XRD patterns of amorphous TiO_2_-20 h and anatase TiO_2_-20 h-450 °C. (**b**) Raman spectra of anatase TiO_2_ nanotubes after annealing. The samples were synthesized in electrolyte with 0.5 wt% NH_4_F and with anodization oxidation time of 20 h.

Figure 4a shows XRD patterns of as-obtained amorphous TiO_2_ and annealed TiO_2_ at 450 °C for 2 h. Before annealing, only the characteristic diffraction peaks of Ti substrate at 34.9, 38.2, 40.0, 52.8, 62.8, 70.5, 76.0, 82.1 and 86.6° existed. After annealing, the characteristic diffraction peaks at 25.2, 36.9, 42.9, 53.8, 54.9, 68.6 and 74.9° belonged to the anatase TiO_2_ phase. The TiO_2_ nanotubes obtained by anodizing for 2, 5, 10, and 20 h had the same crystal structure. The results show that oxidation time had no influence on the crystal phase of samples. Furthermore, Raman spectra were performed to study their structures (Figure 4b). According to group theory analysis of anatase TiO_2_, there were six optical vibration modes of anatase TiO_2_ with Raman activity, which were one A_1g_, two B_1g_, and three E_g_ modes respectively. As shown in Figure 4b, five peaks of anatase TiO_2_ were observed. The peak positions and vibration modes were 140 cm^−1^, E_g1_; 193 cm^−1^, E_g2_; 391 cm^−1^, B_1g(1)_; 511 cm^−1^, A_1(g)_ + B_1g(2)_, and 633 cm^−1^, E_g3_, respectively. This result is consistent with the previously reported pure anatase TiO_2_ structure [37].

**Figure 5:**
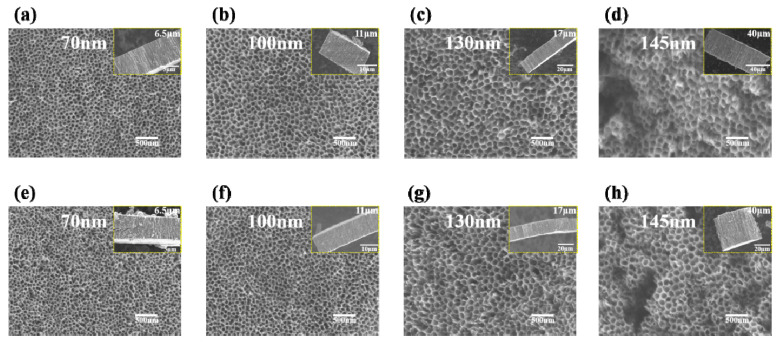
SEM images of top view of TiO_2_ nanotube arrays before (**a**–**d**) and after (**e**–**h**) annealing with different oxidation times in electrolyte with 0.2 wt% NH_4_F: (**a**,**e**) 2 h; (**b**,**f**) 5 h; (**c**,**g**) 10 h; (**d**,**h**) 20 h. Inserts show side view of TiO_2_ nanotube arrays.

Figure 5 shows SEM images of TiO_2_ nanotube arrays before and after annealing with different oxidation times at 0.2 wt% NH_4_F. The diameters and lengths of TiO_2_ nanotubes were: 70 nm and 6.5 μm in 2 h oxidation (Figure 5a); 100 nm and 11 μm in 5 h oxidation (Figure 5b); 130 nm and 17 μm in 10 h oxidation (Figure 5c); and 145 nm and 40 μm in 20 h oxidation (Figure 5d), respectively. The above changes clearly demonstrate the transformation process from nanopores to nanotubes. Compared with amorphous nanotubes, the nozzles of anatase nanotubes collapsed slightly (Figure 5e–h), but the tube diameters and lengths kept the original values, which indicates that TiO_2_ nanotubes obtained by anodic oxidation method have good thermal stability.

**Figure 6:**
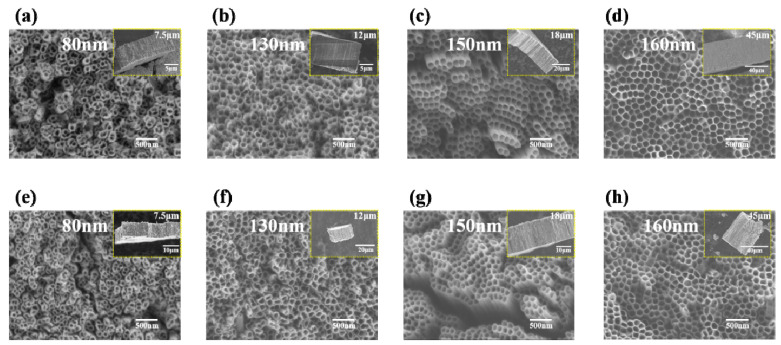
SEM images of top view of TiO_2_ nanotube arrays before (**a**–**d**) and after (**e**–**h**) annealing with different oxidation times in electrolyte with 0.5 wt% NH_4_F: (**a**,**e**) 2 h; (**b**,**f**) 5 h; (**c**,**g**) 10 h; (**d**,**h**) 20 h. Inserts show side view of TiO_2_ nanotube arrays.

Figure 6 demonstrates the change of nanotubes under the electrolyte of 0.5 wt% NH_4_F. The diameters and lengths of TiO_2_ nanotubes were: 80 nm and 7.5 μm in 2 h oxidation (Figure 6a); 130 nm and 12 μm in 5 h oxidation (Figure 6b); 150 nm and 18 μm in 10 h oxidation (Figure 6c); and 160 nm and 45 μm in 20 h oxidation (Figure 6d), respectively. After annealing, nozzles collapsed slightly but the tube diameters and lengths remained the same. Similarly, the morphology of the nanotubes after annealing also kept the same (Figure 6e–h). What is exciting is that in comparison with all the above samples, the nanotube arrays obtained by anodizing at 0.5 wt% NH_4_F for 10 h showed a high-ordered nanotube array.

**Figure 7:**
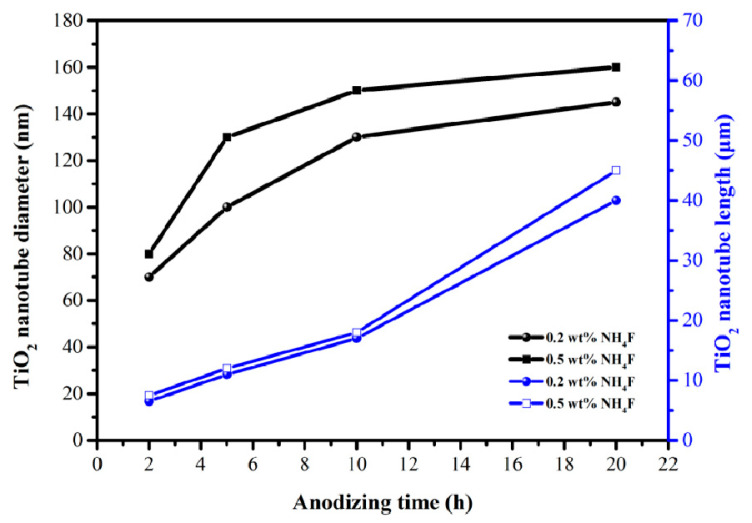
Comparison of TiO_2_ nanotube diameters and lengths obtained under different anodizing times in electrolyte with 0.2 and 0.5 wt% NH_4_F.

The concentration of F^−^ ions can affect the morphology and length of the nanotube. Figure 7 exhibits the changes in the diameter and length of nanotubes with different oxidation times under two different F^−^ concentrations. In this work, the diameter and length of the nanotubes were positively correlated with F^−^ ion concentration and oxidation time. When the F^−^ ion concentration was suitable, more pores were chemically etched uniformly and synchronously on the entire oxide layer surface. An excessively high fluoride ion concentration led to the rapid formation of soluble complex ions [TiF_6_]^2−^, thereby inhibiting the formation of an oxide layer and further hindering the growth of nanotubes. Low F^−^ ion concentration resulted in the insufficient dissolution of the formed barrier layer, and the formation of a dense oxide layer [38,39]. The growth rate of nanotube diameter gradually decreased with the increase of oxidation time, while the increase in nanotube length showed an opposite trend. This was caused by the gradual decrease of the dissolution rate between the nanotubes, and more fluoride ions were used to push the nanotubes toward the titanium substrate. Obviously, the growth and dissolution of the nanotubes had not yet reached an equilibrium, which means that the nanotubes would continue to grow as the oxidation time increased.

Owing to its sufficient capacity and higher lithiation potential (~1.6 V vs. Li/Li^+^), TiO_2_ is often considered as a suitable anode material [28,40,41]. The electrochemical reaction of insertion/extraction of Li^+^ in TiO_2_ nanotube electrodes is as follows:

TiO_2_ + xLi^+^ + xe^−^ ↔ Li_x_TiO_2_ (1)

**Figure 8:**
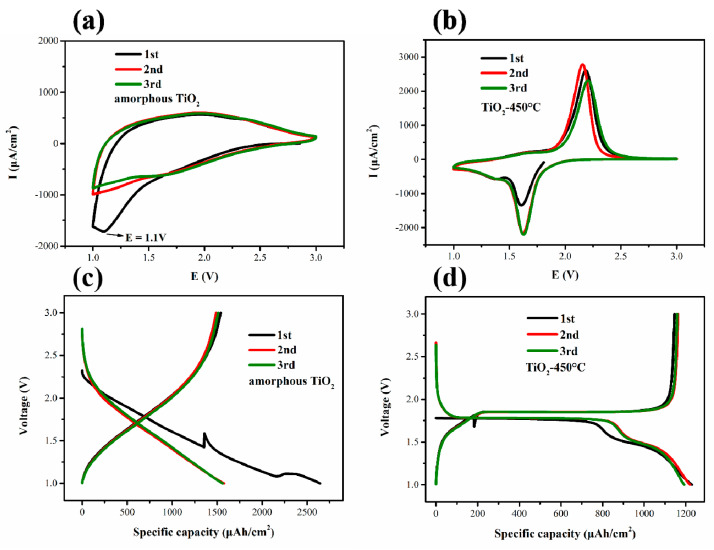
CV curves of amorphous TiO_2_-20 h (**a**) and anatase TiO_2_-20 h-450 °C (**b**) at a sweep rate of 0.2 mV/s. The first three cycles charge/discharge curves of amorphous (**c**) and anatase (**d**) TiO_2_ nanotubes. The voltage range was between 1.0 and 3.0 V, and the constant charge and discharge current was 100 μA/cm^2^. The samples were synthesized in electrolyte with 0.5 wt% NH_4_F and anodization time of 20 h.

Figure 8a,b reveals CV curves of amorphous and anatase TiO_2_ nanotubes. For amorphous TiO_2_ nanotubes, there is a reduction peak around 1.1 V, which indicates that part of the TiO_2_ has undergone a phase change to form a cubic phase of Li_2_Ti_2_O_4_ [42], and it is only visible in the first cycle, proving that it is irreversible. Amorphous TiO_2_ nanotube has mainly displayed pseudocapacitive behavior [43,44]. For anatase TiO_2_ nanotubes, a pair of redox peaks appear around 1.6 and 2.1 V, respectively. The redox process (Ti^4+^ ↔ Ti^3+^) is accompanied by Li^+^ insertion/extraction into the oxide structures, which represents the transition from lithium-poor phase Li_x_TiO_2_ to orthorhombic lithium titanite (Li_~0.55_TiO_2_) [45].

Figure 8c,d shows that charge–discharge curves of amorphous TiO_2_ nanotubes were sloping with no voltage plateau, while the curves of anatase TiO_2_ nanotubes had two obvious plateaus at 1.78 and 1.85 V, which were consistent with CV curves. This result indicates that no two-phase reaction occurred in the amorphous TiO_2_ nanotube electrode. The plateaus in anatase TiO_2_ nanotube electrode can be attributed to the insertion and extraction of Li^+^ from tetrahedral and octahedral positions [46]. Amorphous TiO_2_ nanotubes undergo large irreversible first discharge capacity loss, contributing to irreversible decomposition of part of the electrolyte, and changes of initial oxide morphology, and stoichiometric ratio. Anatase TiO_2_ nanotube electrode showed stable charge–discharge for the initial three cycles, owing to its stable crystal structure. After the second cycle, the battery no longer had a significant loss of discharge capacity, that is, it entered a stable redox stage.

**Figure 9:**
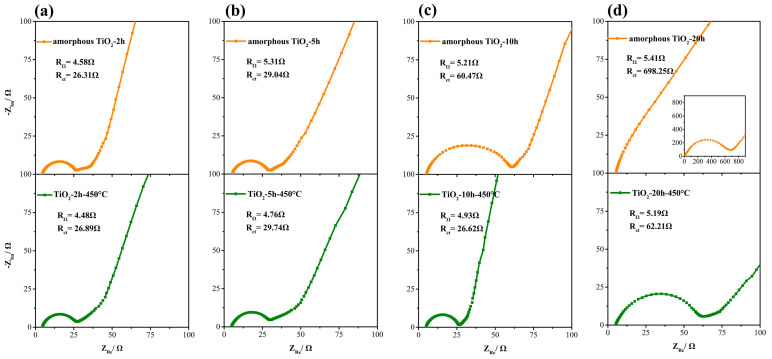
Nyquist plots of amorphous TiO_2_-20 h and anatase TiO_2_-20 h-450 °C anodes under different oxidation times: (**a**) 2 h; (**b**) 5 h; (**c**) 10 h; (**d**) 20 h. The samples were synthesized in electrolyte with 0.5 wt% NH_4_F and anodization time of 20 h.

The kinetic characteristics of TiO_2_ nanotube anodes were analyzed by electrochemical impedance spectroscopy (EIS). Figure 9 shows the Nyquist plots of the amorphous and anatase TiO_2_ anodes prepared with different oxidation times, where R_Ω_ and R_ct_ correspond to electrolyte resistance and interface charge transfer resistance, respectively [47,48]. R_Ω_ of amorphous and anatase TiO_2_ was around 5 Ω, and the value of anatase TiO_2_ was slightly smaller than that of amorphous TiO_2_. The R_ct_ values of amorphous TiO_2_ increased greatly with the increase of the oxidation time, especially when the oxidation time was greater than 5 h. In contrast, the values of anatase TiO_2_ were relatively stable. The results confirm that TiO_2_ with stable crystal structure can display better kinetic performance.

**Figure 10:**
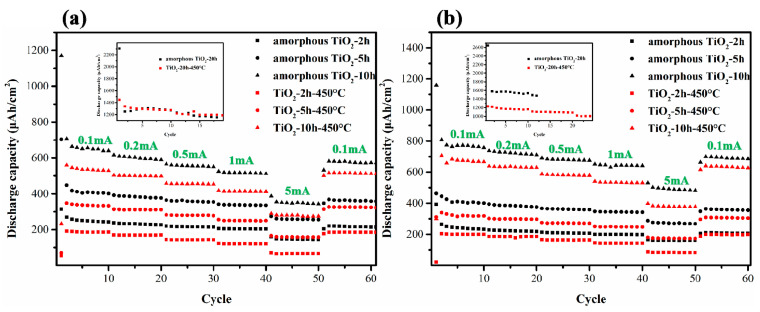
Cycling stability and rate performances of amorphous and anatase TiO_2_ nanotubes with different anodizing times (2, 5, 10 h (inserts: 20 h)) under different current densities and different NH_4_F contents in TiO_2_ nanotubes growth: (**a**) 0.2 wt%, (**b**) 0.5 wt%.

Figure 10 depicts the rate performance and cycling stability of amorphous and anatase TiO_2_ nanotube electrodes. The areal capacities of amorphous TiO_2_ are higher than those of anatase TiO_2_, which is due to the high defects, loose structure, and disorder of amorphous TiO_2_ [30]. For example, reversible capacity of amorphous TiO_2_ is 1350 μAh/cm^2^, while the same value of anatase TiO_2_ is 1240 μAh/cm^2^ for samples obtained under 0.2 wt% NH_4_F and 20 h oxidation time, which is higher than TiO_2_ nanotube foam with areal capacity of 507 μAh/cm^2^ at 50 μA/cm^2^ [30]. In comparison, the rate performance of anatase TiO_2_ electrodes under different current densities was more stable than that of amorphous TiO_2_. Therefore, as lithium-ion battery anodes, anatase TiO_2_ nanotubes show more stable electrochemical performance than amorphous phase. The areal capacities increased with prolonging oxidation time and F^−^ ions concentration. With the increase of oxidation time and F^−^ ions concentration, longer nanotubes were formed in Ti foil, contributing to enhanced areal capacities. As shown in Table 1, with increase of the diameter and length of TiO_2_ nanotubes, the specific area capacities also increased. The increased tube diameter can increase the contact area between the electrode and the electrolyte, while longer TiO_2_ nanotubes can increase the amount of active materials per unit area. Therefore, TiO_2_ nanotubes with larger tube diameter and longer tube length show better electrochemical performances.

## 4. Conclusions

In this work, we studied the relationship between electrochemical performance and crystallographic structure of TiO_2_ and geometry of as-formed TiO_2_ nanotube arrays. Highly ordered TiO_2_ nanotube arrays with a tube diameter of 70–160 nm and length of 6.5–45 μm were grown by anodic oxidation of Ti foil. With the studied growth mechanism, the diameter and length of TiO_2_ nanotubes could be adjusted by controlling oxidation time and F^−^ concentration. The formation of [TiF_6_]^2−^ complexes led to the chemical dissolution of formed TiO_2_ at oxide/electrolyte interface, which favors the growth of nanotubes and increase of tube diameter. The higher the F^−^ concentration, the larger the tube diameter. The tube length was related to the total electrical charge. Thus, the longer the reaction time, the longer the tube length. F^−^ concentration did not affect the increase trend of tube length, but only increased the TiO_2_ tube diameter. Serving as Li-ion battery anodes, reversible capacities of amorphous TiO_2_-20 h and anatase TiO_2_-20 h-450 °C were 1350 and 1240 μAh/cm^2^, respectively. The higher areal capacity of amorphous TiO_2_ was due to the high defects and loose structure of the amorphous phase. However, anatase TiO_2_ nanotubes showed better rate performance owing to their stable crystallographic structure. Anatase TiO_2_ nanotubes with longer tubes showed higher areal capacity and stable cycling performances. The increased tube diameter increased the contact area between the electrode and the electrolyte, and longer TiO_2_ nanotubes increased the amount of active materials per unit area. The anodic oxidation method can be a facile nanotechnology tool to synthesize other 1D nanotube metal oxides, i.e., Nb_2_O_5_, Fe_2_O_3_, ZnO, CuO etc.

## Figures and Tables

**Table 1:** Relationship between the second discharge capacity and the diameter/length of the nanotubes.

Anodization Electrolyte	Samples	Nanotube Diameter (nm)	Nanotube Length (μm)	2nd Discharge Capacity (μAh/cm^2^)
0.2 wt% NH_4_F	TiO_2_-2 h	70	6.5	269.1
	TiO_2_-5 h	100	11	448.2
	TiO_2_-10 h	130	17	705.8
	TiO_2_-20 h	145	40	1251.1
	TiO_2_-2 h-450 °C	70	6.5	190.4
	TiO_2_-5 h-450 °C	100	11	347.3
	TiO_2_-10 h-450 °C	130	17	558.8
	TiO_2_-20 h-450 °C	145	40	1336.9
0.5 wt% NH_4_F	TiO_2_-2 h	80	7.5	264.1
	TiO_2_-5 h	130	12	444.2
	TiO_2_-10 h	150	18	808.3
	TiO_2_-20 h	160	45	1576.1
	TiO_2_-2 h-450 °C	80	7.5	203.2
	TiO_2_-5 h-450 °C	130	12	339.5
	TiO_2_-10 h-450 °C	150	18	706.3
	TiO_2_-20 h-450 °C	160	45	1220.1

## Data Availability

Data is contained within the article material.
